# Progress in Gynæcology

**Published:** 1901-04-06

**Authors:** 


### Progress in Gynæcology.

(*Continued from page* 421.)

**Uterine Displacements.**—Charles A. L. Reed[7] recommends intraperitoneal shortening of the round ligaments for retrodisplacements of the uterus. He makes a letter-of-S fold in the ligaments, and stitches them thus folded to the parietal peritoneum along the line of Poupart's ligament. He thinks, however, that the parietal fixation of the folded ligaments is not absolutely necessary for holding the uterus in its normal position. He has devised an ingenious instrument, viz., a pair of forceps with four flat projecting prongs, by which the ligament is seized, twisted, and held in position until the necessary sutures are applied; these are inserted as follows: an interrupted one fixing the inner loop of ligament to the corner of the uterus, and a similar one fixing the outer fold of the ligament; a continuous suture is then passed between the prongs of the fixation forceps, its ends being obliquely tied after the instrument is withdrawn. He points out that in many cases of retroflexion and anteflexion there is present an atrophy of the concave, and hypertrophy of the convex wall of the uterus at the point of flexion, and this elongated and hypertrophied wall often offers a persistent resistance to the uterus assuming its normal axis; to overcome this he recommends the removal of a cuneiform segment from the hypertrophied wall. An operation which Thiriar calls cuneohysterectomie, and which is applicable in either anterior or posterior flexions. Ferrari[8] reports four cases of retrodeviation of the uterus in which he adopted the following method of treatment with very good results. The bowels having been well opened for two days before the operation, and the usual disinfection of the vagina carried out, a transverse incision is made in the posterior vaginal fornix, and the pouch of Douglas opened. The finger is then introduced, adhesions broken down, and the uterus drawn upwards and forwards into a good position. The pouch of Douglas is now lightly packed with iodoform gauze to the level of the internal os, and the uterus forced upwards and backwards against the sacral promontory by a tampon in the vagina. The bladder must be kept empty for several days by a self-retaining catheter, and the bowels undisturbed for ten days. The gauze is left in the pouch of Douglas for from two to four days according to the amount of exudation. The vaginal tampon is renewed every day for a fortnight, the vaginal wound being washed with an antiseptic every time the dressing is changed. The author says the simplicity of the method, its rapidity and absence of danger are striking. A. Lapthorn Smith,[9] writing on the treatment of procidentin uteri in elderly women, advocates the adoption of ome operative procedure in all cases when the patient is unable to wear a pessary, and says that the prevailing notion that it is hardly worth while to operate on women of 60 or 70 years of age, is responsible for much unnecessary suffering. When the uterus is small and not far enough out of the body to have become ulcerated, he recommends ventrofixation. The vaginal outlet is then narrowed by an anterior and posterior colporrhaphy. When the uterus, however, is long it is better to amputate all but the upper two inches of it, and stitch the vagina to the upper half. Although the writer has removed many prolapsed uteri, he thinks it is better in the majority of cases to perform ventrofixation, and amputate the cervix, because the bladder and vagina are more effectually drawn up by this procedure than by complete removal of the uterus.

**The Pathology of Tubal Pregnancy.**—Maximilian Herzog,[10] writing on this subject, says he discards the theory that inflammatory disease of the tube is a cause of tubal pregnancy. The factors which he considers of most importance are congenital anomalies of the tubes, and an unduly marked participation of the tubal mucosa in menstruation. The early fœtal placenta of tubal pregnancy does not differ from one of the same age in uterine gestation. The blood in the vessels of the chorionic villi is of course fœtal blood, containing nucleated red corpuscles and no leucocytes; that in the intervillous spaces is maternal, containing the ordinary non-nucleated red corpuscles and the due proportion of leucocytes. A decidua scrotina is certainly formed in tubal pregnancy. Under the stimulus of the developing ovum in the tube, the cells of the connective tissue in the mucosa become enlarged, and opposite the site of the ovum are found to closely resemble the decidual cells in the uterine serotina, being large, oval or polygonal, with large round or oval vesicular nuclei. This arrangement, however, is seen in a few cases only, pathological changes occurring so early that the original condition is soon obscured. The earliest pathological changes occur in the tubal structures lying outside the mucosa. There may be slight hypertrophy of the muscular fibres of the middle coat of the tube, but more frequently there is atrophy, the bundles of the fibres being pushed apart, and the interstices between them either filled with loose connective tissue and leucocytes or with an œdematous infiltration. An œdematous infiltration of the subperitoneal layer of the tube soon follows. The opening up of the maternal blood sinuses by the syncytium is enormously exaggerated in tubal pregnancy, and it is this that causes the hæmorrhage both into the tube wall and into the intervillous spaces. The hæmorrhage from the decidua into the intervillous spaces causes the death of the embryo by damaging the villi, and thus interfering with its nutrition. Rupture or tubal abortion soon follow after the death of the embryo.

**Hysterectomy.**—Dr. A. A. Warden[11] published eighteen cases of abdominal hysterectomy in which Dr. Doyen's technique was followed. In Dr. Doyen's hands this method gives the very low mortality of from 2 to 4 per cent. The following is a brief résumé of the operation: The patient is placed in the Trendelenburg position, the abdomen opened and the tumour drawn out above the pubes. The posterior vaginal cul-de-sac is opened from the abdomen by scissors cutting upon curved forceps introduced per vaginum by an assistant; the incision is extended a little on either side, and the cervix firmly seized by its posterior lip with strong catch forceps and drawn well up; it is then freed by cuts with the scissors to the right and left until it is completely circumcised. It is then drawn well up, separating itself from the bladder. The uterus is now only attached by its lateral vascular connections, and these are rapidly divided, the uterine and ovarian vessels being seized with pressure forceps and ligatured. The right and left appendages are now ligatured and removed, any bleeding vessels ligatured and the pelvic cavity sponged out. The retrovesical peritoneum is then united to that of Douglas's pouch, completely closing the opening into the vagina. The abdominal wound is then closed, and no drainage as a rule employed. Dr. A. Goldspohn[12] reports two cases of intestinal obstruction following vaginal hysterectomy. In one case the abdomen was reopened eighty-four hours after operation, and a loop of intestine found adherent in the bottom of the cul-de-sac, when it appeared to have been compressed by the gauze drain, but it was not completely occluded and was easily raised. General peritonitis rapidly set in, and the patient died thirty-six hours after the ventral operation; in the other case the patient's condition was very satisfactory until the ninth day, when she was seized with severe abdominal pain, accompanied with distension and vomiting. The abdomen was opened on the following day, when two loops of the ileum with a mass of omentum were found adherent to the posterior edge of the vaginal wound, and were severely kinked and drawn upon: the adherent bowel was freed, the peritoneum sponged out and drainage secured by means of a gauze drain in the vagina, and three gauze drains from different points in the abdomen. The bowels were opened satisfactorily six hours after the operation and the patient made a complete recovery. Dr. John Aspell[13] records a case of tetanus following abdominal hysterectomy for a uterine fibroid. Convalescence was uneventful until the tenth day, when slight twitching of the muscles of the face and left arm were noticed. An hour later there was rigidity of the jaw, and the patient died during a spasm thirty-six hours after the onset of the disease. She was treated with the antitetanic serum from the first. One of the nurses who assisted at the operation had a patient recovering from tetanus under her care in the ward.

[7] Med. Rec., Oct. 6, 1900.. [8] Brit. Gyn. Jour., Nov. 1900. [9] The Canadian Pract. and Rev., Oct. 1900. [10] Amer. Jour. of Obstet., Aug. 1900. [11] Lancet, Oct. 13, 1900. [12] Med. Rec., Sept. 18, 1900. [13] Amer. Jour. of Obstet., Dec. 1900.

